# Integrated mosquito control in Matatang village, Northern Zhejiang, China: an effectiveness assessment

**DOI:** 10.3389/fpubh.2025.1628116

**Published:** 2025-07-31

**Authors:** Song Guo, Jian Huang, Ying Liu, Rong Zhang, Jiangping Ren, Xuguang Shi, Jimin Sun

**Affiliations:** ^1^Zhejiang Key Lab of Vaccine, Infectious Disease Prevention and Control, Zhejiang Provincial Center for Disease Control and Prevention, Hangzhou, Zhejiang, China; ^2^Jiashan Center for Disease Control and Prevention, Jiaxing, Zhejiang, China

**Keywords:** mosquito control, effectiveness assessment, integrated vector management, health education, rural village

## Abstract

Mosquito nuisance and disease transmission have become significant challenges in rural development and tourism. From 2018 to 2021, Matatang village in Jiaxing City, Zhejiang Province, implemented an integrated mosquito control program centered on environmental management and health education. This study evaluated the effectiveness of mosquito control in Matatang village by assessing mosquito abundance, villagers’ knowledge, behaviors, perceptions regarding mosquito control, and satisfaction rates. Mosquito abundance data were collected during the intervention phase (2018) and maintenance phase (2021), including adult and larval mosquito surveillance. Villagers’ knowledge, behaviors, perceptions, and satisfaction were assessed through surveys during the maintenance phase (2021). A significant decline in abundance was observed in all mosquito species between monitoring periods. Surveillance results showed that the mean adult mosquito index in Matatang village was 9.35 ± 9.82 in 2018 and 1.95 ± 1.49 in 2021, compared to 6.45 ± 4.46 in the control village in 2021. The mean larval mosquito index in Matatang village was 36.00 ± 39.19 in 2018 and 9.50 ± 4.11 in 2021, whereas the control village had a mean larval index of 35.50 ± 27.25 in 2021. Statistical analysis revealed significant differences in monthly adult and larval mosquito indices between Matatang village and the control village in 2021 (*p* < 0.05). Survey results indicated significantly higher knowledge levels of basic mosquito-related information among Matatang village residents compared to the control village. Notably, awareness of mosquito breeding sites exceeded 80% in Matatang village, while remaining below 25% in the control village, with this difference demonstrating statistical significance (*p* < 0.001). Behavioral practices such as eliminating stagnant water, cleaning ditches, and regularly changing water for hydroponic plants were also significantly more prevalent in Matatang village. Moreover, 73.33% of Matatang villagers expressed satisfaction with local mosquito control efforts, compared to only 10.00% in the control village (*χ^2^* = 24.754, *p* < 0.001). This study demonstrates that an integrated mosquito control model emphasizing environmental management and health education can foster long-term self-management and proactive maintenance among villagers. Such an approach not only sustainably reduces mosquito abundance but also improves rural living conditions, highlighting its critical public health significance.

## 1 Introduction

Medically relevant mosquitoes belong to the order Diptera and family Culicidae (1, 2), such as *Aedes albopictus* (3) and *Culex quinquefasciatus* (4). As important viral reservoirs and vectors (5), they are closely associated with humans. Beyond causing nuisance through biting and blood-feeding, they can transmit various diseases, including malaria, dengue fever, Chikungunya fever, yellow fever, and Zika virus disease (6–10). With frequent trade and tourism exchanges between China and regions like Southeast Asia and Africa, the annual number of imported cases of mosquito-borne diseases continues to rise (11, 12). The frequency and geographic spread of local outbreaks of mosquito-borne diseases such as dengue fever and Chikungunya fever have significantly increased (13, 14). Due to the lack of effective vaccines and specific treatments (15, 16), mosquito control is widely recognized as a critical measure for preventing and controlling mosquito-borne diseases like dengue fever (17).

Zhejiang Province, located in the subtropical coastal region of eastern China, experiences year-round humid and rainy conditions, which create an ideal environment for mosquito breeding (18). In recent years, urban and rural sanitation campaigns have flourished across the province, leading to significant improvements in environmental conditions. However, mosquito abundance remains high in some rural areas, making mosquito nuisance and disease transmission a major challenge for the construction of beautiful villages and the development of rural tourism (19, 20). Mosquito reproduction relies on aquatic environments (21), yet rural areas face underdeveloped sanitation infrastructure, including inadequate construction of sanitary toilets, waterways, and drainage systems (22). Improper disposal of household waste, sewage, and livestock manure, coupled with villagers’ unhygienic practices like outdoor water storage and improper waste disposal (23), further aggravate the situation. Compounded by insufficient health education and scientific knowledge, as well as weak hygiene awareness and self-care practices among rural residents (24, 25), these factors contribute to rampant mosquito breeding in poorly managed rural environments. Traditional mosquito control methods rely heavily on chemical pesticides. While these can rapidly reduce mosquito abundance in the short term, they lack sustainability—mosquito populations often rebound quickly after pesticide efficacy diminishes (26, 27). Moreover, excessive pesticide use poses potential threats to the environment and human health (28), as well as the risk of insecticide resistance (29). Therefore, implementing sustainable mosquito control measures in rural areas is essential (30).

In 2018, Matatang village in Jiaxing City, Zhejiang Province, launched a pilot program for sustainable mosquito control centered on environmental management and health education. Under the guidance of professionals from the Center for Disease Control and Prevention (CDC), the village committee formulated a mosquito control plan and established relevant management protocols. Efforts included mobilizing villagers through awareness campaigns, conducting health education lectures, and investigating mosquito breeding sites, followed by the progressive implementation of integrated mosquito control strategies. Through targeted intervention activities, the village environment underwent significant beautification, leading to a substantial reduction in mosquito breeding sites and a continuous decline in mosquito abundance. Concurrently, Matatang village residents demonstrated significantly heightened initiative in autonomously implementing science-based vector control measures, transforming the community into a health-optimized exemplar of a “Beautiful Village”.

In this study, we evaluated the effectiveness of integrated mosquito control measures in Matatang village, summarizing successful strategies for rural mosquito management. The findings hold significant implications for improving rural health standards, promoting local economic development, and informing public health policy formulation.

## 2 Materials and methods

### 2.1 Description of study area

**Figure 1:**
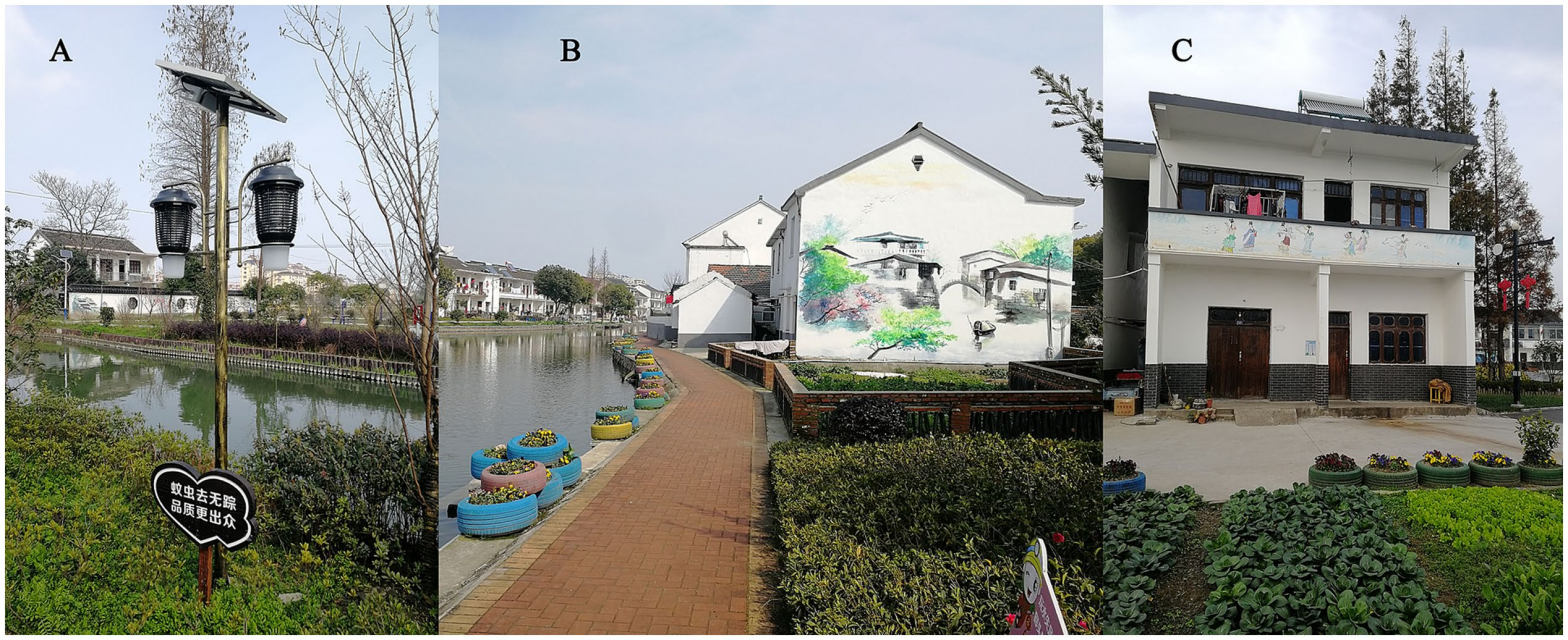
The environment of Matatang village. **(A)** Solar-powered mosquito light trap installed along the riverbank. **(B)** Waste tire repurposed as a flower pot. **(C)** Neatly organized farmhouse courtyard free of mosquito breeding sites.

Matatang village is located in the northern plains of Zhejiang Province, with an average annual temperature of approximately 15.5°C and an average annual precipitation of around 1,100 mm. The village encompasses a total land area of 1.36 square kilometers, consisting of six hamlets, and maintains a registered resident population of approximately 1,300 individuals. The primary site for mosquito control was Dongshengbang hamlet, which has approximately 60 households and a population of nearly 180 people. In recent years, Matatang village has vigorously developed its collective economy and actively improved the village living environment, achieving significant results (Figure 1).

### 2.2 Integrated mosquito control measures

The integrated mosquito control measures implemented in Matatang village was concisely described across four key components: organizational management, vector control interventions, health education initiatives, and sustainability mechanisms.

### 2.3 Study design and control selection

**Figure 2:**
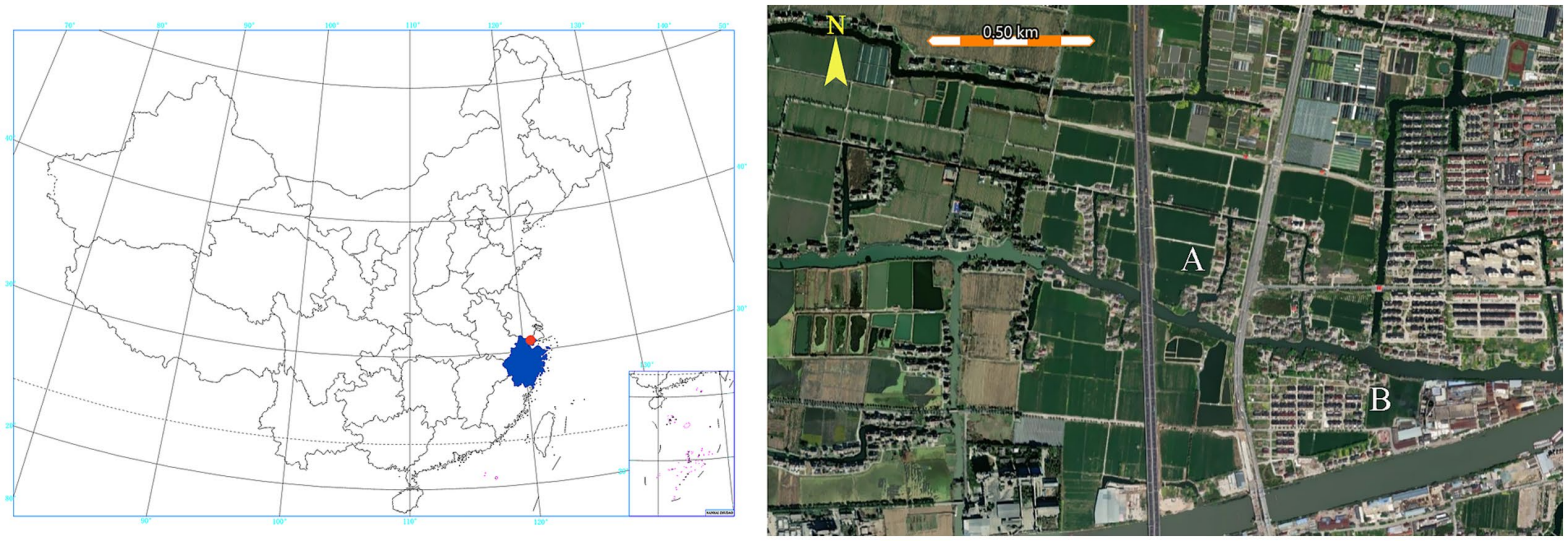
Mosquito control and surveillance area in this study. The study area (red dot) is located in the northern part of Zhejiang Province (blue-shaded area) (left panel). Matatang village (A) and the control village (B) are separated by a river (right panel).

The evaluation of effectiveness was conducted in three aspects: mosquito abundance, villagers’ knowledge, behaviors and perceptions regarding mosquito control, and villager satisfaction rate. Mosquito abundance data were derived from surveillance during the intervention phase (2018) and maintenance phase (2021) of control activities. Results on villagers’ knowledge, behaviors and perceptions and satisfaction rates were obtained through surveys conducted during the maintenance phase (2021). Hongfu village, situated south of Matatang village, was selected as the control village. It shares comparable geographical conditions, cultural practices, and socioeconomic status with Matatang village, with a documented area of 5.97 square kilometers and a population of 3,650 residents. No organized mosquito control interventions have been implemented in Hongfu village, where residents maintain exclusively traditional mosquito management practices. The control site was positioned over 100 meters from Matatang village, separated by a natural river barrier measuring 20 to 30 meters in width (Figure 2).

### 2.4 Mosquito abundance surveillance

From April to November, surveillance of adult mosquito abundance and larval mosquito abundance was conducted once a month within the village. Adult mosquito abundance surveillance involved deploying five CO_2_-baited mosquito light traps per village, strategically positioned in residential areas and public spaces such as green belts, pond banks, and riverbanks. The light trap hanging points were selected to be away from interfering light sources and sheltered from wind, with the height standardized at 1.5 meters above ground level. Monitoring commenced 1 h before sunset and concluded at dawn the following day, with the captured female mosquitoes counted and recorded. Adult mosquito abundance was expressed as the adult mosquito index, which represented the average number of female mosquitoes captured per light trap per night. For larval mosquito abundance surveillance, various types of stagnant water in containers, puddles, and drainage system wells were inspected for the presence of mosquito larvae or pupae. The external environment of 30 square meters was considered equivalent to one household. A total of 50 households per village were monitored for larval mosquito abundance. Larval mosquito abundance was expressed as the larval mosquito index, which represented the number of positive water containers per 100 households in both indoor and outdoor environments.

### 2.5 Cross-sectional villager survey

In each village, 30 households were randomly selected to conduct a questionnaire survey on villagers’ knowledge, behaviors and perceptions regarding mosquito control as well as their satisfaction level. The survey was conducted in Dongshengbang hamlet (60 households), the core intervention area of Matatang village, with 50% household sampling coverage to ensure population representativeness. The survey content included general demographic information, basic knowledge about mosquitoes, commonly used mosquito control measures, demand for governmental support, knowledge acquisition pathways, and satisfaction rate with mosquito control in the village. Questions 1 to 4 (Q1–Q4) were single-choice questions assessing basic knowledge of mosquito. The statistical analysis calculated the percentage of respondents who selected the correct answer for each question out of the total number of participants. Questions 5 to 7 (Q5–Q7) were multiple-choice questions evaluating mosquito control behaviors and perceptions. The statistical analysis determined the percentage of respondents who selected each option for every question relative to the total number of participants. The villagers’ satisfaction rate was divided into three levels: “Satisfied” indicated very few mosquitoes in the village with minimal biting; “Neutral” meant occasional mosquito nuisance in some areas; and “Dissatisfied” represented abundant mosquitoes with severe biting problems. The satisfaction rate was calculated as the proportion of respondents selecting “Satisfied” among all surveyed participants (Q8).

### 2.6 Statistical analysis

Statistical analyses were performed using Excel and Python 3.13.3 (31). The mean mosquito index was presented as mean ± standard deviation (SD). Welch’s t-test was used for pairwise comparisons of mosquito abundance data, while Pearson’s chi-square test or Fisher’s exact test was primarily applied for pairwise comparisons of questionnaire survey data. *p* < 0.05 was considered statistical significance.

## 3 Results

### 3.1 Integrated mosquito control measures

Village management personnel were responsible for community mobilization, program formulation, and implementation of interventions within the integrated mosquito control framework. The integrated approaches prioritized environmental management, focusing on the elimination and modification of breeding habitats, supplemented by physical, biological, and chemical control measures. Before the end of March, villagers were organized to remove potential breeding sites and target early spring mosquitoes—an intervention proven to significantly enhance annual mosquito control efficacy. Area-based management was implemented in phases, with priority given to environmental remediation in key zones, including building rooftops, indoor and peri-domestic areas, abandoned constructions and construction sites, bamboo groves and greenbelts, as well as ditches and ponds. Subsequent interventions, guided by monitoring results, addressed vegetable plots, rice fields, drainage channels, and stormwater wells, complemented by physical (solar-powered mosquito light traps, insect screens on doors/windows) biological (introduction of small larvivorous fish) and chemical (application of slow-release larvicides and targeted adulticide spraying) measures to minimize mosquito proliferation. Public health professionals conducted regular health education and field guidance on evidence-based mosquito control for villagers, alongside monthly entomological surveillance. Findings informed targeted interventions to address identified issues. Starting in 2019, village volunteers and residents initiated routine environmental inspections to identify and eliminate newly formed breeding sites, establishing a long-term maintenance mechanism.

### 3.2 Mosquito abundance surveillance data

Mosquito abundance surveillance data serve as the most direct indicator for evaluating vector control effectiveness. Surveillance of mosquito abundance was conducted in Matatang village in 2018 and 2021, with a control village surveyed in 2021, providing both self-controlled and blank-controlled comparisons for Matatang’s mosquito monitoring results. Different methodologies were employed to assess adult and larval mosquito abundance, with larval surveillance resembling the Breteau Index method but without species differentiation.

The predominant adult mosquito species detected were *Cx. quinquefasciatus*, *Culex tritaeniorhynchus*, *Ae. albopictus*, *Anopheles sinensis*, and *Armigeres obturbans*. *Cx. quinquefasciatus* and *Ae. albopictus* were the dominant mosquito species, comprising the highest proportion of captured specimens. Entomological monitoring in Matatang village revealed peak captures for both species in May 2018 (*Cx. quinquefasciatus*: 101; *Ae. albopictus*: 31). By 2021, peak *Cx. quinquefasciatus* captures shifted to June (*n* = 15), while *Ae. albopictus* peaked in June and July (*n* = 5 each). A significant decline in abundance was observed for both species between monitoring periods. In Matatang village, the mean adult mosquito index in 2018 was 9.35 ± 9.82, peaking in May (29.20) and reaching the lowest level in November (0.00). In 2021, the mean index decreased to 1.95 ± 1.49, with the highest value in June (4.80) and the lowest in November (0.40). The control village in 2021 exhibited a mean adult mosquito index of 6.45 ± 4.46, with a maximum in September (13.80) and a minimum in November (1.60). Statistical analysis indicated no significant difference in mean adult mosquito indices between Matatang village in 2018 and 2021 (*t* = 2.107, *p* = 0.071). However, a statistically significant difference was observed between Matatang village and the control village in 2021 (*t* = −2.708, *p* = 0.025), with Matatang’s 2021 mean adult mosquito index being significantly lower than that of the control village (Tables 1–3).

**Table 1:** Surveillance data on adult mosquito abundance in Matatang village during 2018.

Month	Number of light traps deployed	Number of female mosquitoes captured					Total	Adult mosquito index
		*Cx. quinquefasciatus*	*Cx. tritaeniorhynchus*	*Ae. albopictus*	*An. sinensis*	*Arm. obturbans*		
4	5	18	4	8	1	4	35	7.00
5	5	101	5	31	3	6	146	29.20
6	5	60	3	24	0	2	89	17.80
7	5	20	2	27	1	1	51	10.20
8	5	16	2	13	0	2	33	6.60
9	5	2	0	5	0	1	8	1.60
10	5	7	1	4	0	0	12	2.40
11	5	0	0	0	0	0	0	0.00

**Table 2:** Surveillance data on adult mosquito abundance in Matatang village during 2021.

Month	Number of light traps deployed	Number of female mosquitoes captured					Total	Adult mosquito index
		*Cx. quinquefasciatus*	*Cx. tritaeniorhynchus*	*Ae. albopictus*	*An. sinensis*	*Arm. obturbans*		
4	5	4	0	1	1	0	6	1.20
5	5	7	1	4	0	0	12	2.40
6	5	15	3	5	0	1	24	4.80
7	5	10	0	5	1	0	16	3.20
8	5	4	0	0	0	0	4	0.80
9	5	6	0	3	0	1	10	2.00
10	5	4	0	0	0	0	4	0.80
11	5	2	0	0	0	0	2	0.40

**Table 3:** Surveillance data on adult mosquito abundance in the control village during 2021.

Month	Number of light traps deployed	Number of female mosquitoes captured					Total	Adult mosquito index
		*Cx. quinquefasciatus*	*Cx. tritaeniorhynchus*	*Ae. albopictus*	*An. sinensis*	*Arm. obturbans*		
4	5	6	3	3	1	2	15	3.00
5	5	34	4	10	2	7	57	11.40
6	5	26	3	11	1	2	43	8.60
7	5	22	2	6	1	1	32	6.40
8	5	17	0	5	0	0	22	4.40
9	5	43	2	19	1	4	69	13.80
10	5	10	1	1	0	0	12	2.40
11	5	6	0	2	0	0	8	1.60

For larval surveillance, the dominant species belonged to the genera *Culex* and *Aedes*. Matatang village’s mean larval mosquito index in 2018 was 36.00 ± 39.19, peaking in May (116.00) and dropping to zero in November (0.00). In 2021, the mean index declined to 9.50 ± 4.11, with the highest value in September (16.00) and the lowest in November (2.00). The control village in 2021 had a mean larval index of 35.50 ± 27.25, reaching a maximum in September (82.00) and a minimum in November (6.00). Statistical analysis revealed no significant difference in mean larval indices between Matatang village in 2018 and 2021 (*t* = 1.902, *p* = 0.098), whereas a significant difference existed between Matatang village and the control village in 2021 (*t* = −2.669, *p* = 0.031), with Matatang’s mean larval index being significantly lower (Table 4).

**Table 4:** Surveillance data on larval mosquito abundance in Matatang village and the control village during 2018 and 2021.

Month	Number of households inspected	Matatang village (2018)		Matatang village (2021)		Control village (2021)	
		Number of positive water containers	Larval mosquito index	Number of positive water containers	Larval mosquito index	Number of positive water containers	Larval mosquito index
4	50	19	38.00	4	8.00	6	12.00
5	50	58	116.00	6	12.00	20	40.00
6	50	31	62.00	6	12.00	33	66.00
7	50	23	46.00	4	8.00	8	16.00
8	50	5	10.00	4	8.00	10	20.00
9	50	6	12.00	8	16.00	41	82.00
10	50	2	4.00	5	10.00	21	42.00
11	50	0	0.00	1	2.00	3	6.00

### 3.3 General characteristics of the surveyed population

A randomized household survey was conducted in Matatang village and the control village, yielding 60 completed questionnaires. Analysis revealed that respondents were predominantly female household members with thorough knowledge of family circumstances. The majority were aged 40–60 years, had attained education levels no higher than high school, and were primarily engaged in occupations such as farming, homemaking, or industrial labor.

### 3.4 Survey results on villagers’ knowledge, behaviors and perceptions regarding mosquito control

The survey assessed villagers’ knowledge of dengue fever and mosquito control through four key questions. Residents of Matatang village demonstrated significantly higher awareness rates than those in the control village (Q1: *χ^2^* = 9.317, *p* < 0.01; Q2: *χ^2^* = 10.756, *p* < 0.01). However, dengue fever remained poorly understood in both villages, with less than 50% awareness in Matatang village. Most respondents were unfamiliar with government-promoted information on dengue transmission routes and preventive measures. Regarding knowledge of mosquito breeding sites, Matatang village, which received health education and on-site guidance, exhibited substantially higher awareness (>80%) compared to the control village (<25%), with statistically significant differences (Q3: *χ^2^* = 19.288, *p* < 0.001; Q4: *χ^2^* = 26.667, *p* < 0.001) (Table 5).

**Table 5:** Survey results on mosquito control knowledge and behaviors among residents of Matatang village and the control village in 2021.

Survey content		Matatang village (*n* = 30)	Control village (*n* = 30)	*χ^2^*	*p* value
Basic knowledge of mosquito (four single-choice questions)	Q1: Transmission route of dengue fever	12 (40.00%)	2 (6.67%)	9.317	**0.002**
	Q2: Most effective measures for dengue prevention	13 (43.33%)	2 (6.67%)	10.756	**0.001**
	Q3: Potential mosquito breeding sites	24 (80.00%)	7 (23.33%)	19.288	**<0.001**
	Q4: Can stagnant water in tree holes, bamboo tubes, and tires serve as mosquito breeding sites?	25 (83.33%)	5 (16.67%)	26.667	**<0.001**
Q5: Frequently used mosquito control measures (multiple-choice question)	Use of mosquito swatters, mosquito traps, screened doors/windows, and bed nets	25 (83.33%)	26 (86.67%)		1.000
	Application of insect repellents when outdoors	6 (20.00%)	5 (16.67%)	0.111	0.739
	Regular elimination of stagnant water in containers and the environment	22 (73.33%)	4 (13.33%)	21.991	**<0.001**
	Modification and unclogging of drainage ditches	12 (40.00%)	0 (0.00%)	15.000	**<0.001**
	Use of chemical control products (mosquito coils, aerosol insecticides)	26 (86.67%)	22 (73.33%)	1.667	0.197
	Regular water replacement for hydroponic plants	15 (50.00%)	7 (23.33%)	4.593	**0.032**
	No action taken/Unaware of necessary actions	1 (3.33%)	3 (10.00%)		0.612

Bolded *p* values: statistically significant.

The survey results on mosquito control behaviors revealed that villagers in both Matatang village and the control village showed similar preferences for chemical mosquito control products (such as mosquito coils and insecticide sprays; *χ^2^* = 1.667, *p* > 0.05) and physical control measures (such as electric mosquito swatters, mosquito traps, window screens, and bed nets; *p* > 0.05), which are the most traditionally and widely used methods. However, significant differences were observed in environmental modification and elimination of mosquito breeding sites. These measures were less utilized in the control village but had higher adoption rates in Matatang village. Statistically significant differences were found in practices such as regular removal of stagnant water from containers and the environment (*χ^2^* = 21.991, *p* < 0.001), modification and dredging of ditches (*χ^2^* = 15.000, *p* < 0.001), and periodic water replacement for hydroponic plants (*χ^2^* = 4.593, *p* < 0.05). In contrast, the use of repellent products was low in both villages, with no significant difference (*χ^2^* = 0.111, *p* > 0.05) (Table 5).

Regarding government assistance in mosquito control, a higher proportion of villagers in the control village (76.67%) believed that the government should conduct centralized insecticide spraying (*χ^2^* = 8.297, *p* < 0.05). In Matatang village, villagers expressed broader support for multiple approaches, including organized environmental cleanup campaigns, public health education, and centralized spraying. As for sources of mosquito control knowledge, digital platforms (e.g., the internet, Weibo, and Douyin) were slightly more influential than other channels. Notably, Matatang village had significantly higher rates of acquiring knowledge through health education lectures (*χ^2^* = 4.356, *p* < 0.05) and interpersonal communication with peers (*χ^2^* = 7.680, *p* < 0.05) compared to the control village (Table 6).

**Table 6:** Survey results on demand for governmental support, knowledge acquisition pathways, and satisfaction rates regarding mosquito control among residents of Matatang village and the control village in 2021.

Survey content		Matatang village (*n* = 30)	Control village (*n* = 30)	*χ^2^*	*p* value
Q6: Ways the government can assist the public in mosquito control (multiple-choice question)	Area-wide insecticide spraying	12 (40.00%)	23 (76.67%)	8.297	**0.004**
	Community clean-up and environmental management campaigns	13 (43.33%)	7 (23.33%)	2.700	0.100
	Public health education on mosquito control	10 (33.33%)	8 (26.67%)	0.317	0.573
	No assistance required	3 (10.00%)	5 (16.67%)		0.706
Q7: Ways to obtain mosquito control knowledge (multiple-choice question)	Television, radio, newspapers	4 (13.33%)	6 (20.00%)	0.480	0.488
	Internet platforms (e.g., Weibo, Douyin)	11 (36.67%)	9 (30.00%)	0.300	0.584
	Community bulletin boards	9 (30.00%)	7 (23.33%)	0.341	0.559
	Educational pamphlets	8 (26.67%)	6 (20.00%)	0.373	0.542
	Interpersonal communication	9 (30.00%)	1 (3.33%)	7.680	**0.006**
	Health education lecture	11 (36.67%)	4 (13.33%)	4.356	**0.037**
Q8: Your satisfaction level with local mosquito management (single-choice question)	Satisfied	22 (73.33%)	3 (10.00%)	24.754	**<0.001**
	Neutral	7 (23.33%)	9 (30.00%)		
	Dissatisfied	1 (3.33%)	18 (60.00%)		

Bolded *p* values: statistically significant.

### 3.5 Survey results on villager satisfaction rates

Among surveyed residents in Matatang village, only 3.33% expressed dissatisfaction with local mosquito control measures, while 73.33% reported satisfaction—a rate significantly higher than that in the control village (10.00%; *χ^2^* = 24.754, *p* < 0.001) (Table 6). The data indicated statistically significant differences in satisfaction levels between the two villages. Residents of Matatang village reported high satisfaction with the effectiveness of scientific mosquito control interventions, noting a substantial reduction in mosquito nuisance in daily life, with minimal bites or disturbances experienced.

## 4 Discussion

This study evaluated the integrated mosquito control effectiveness in Matatang village between 2018 and 2021. The results demonstrated that through the implementation of sustainable mosquito control measures, the mosquito abundance in Matatang village has been maintained at relatively low levels over the long term. Most villagers have become familiar with mosquito control concepts and methodologies, incorporating breeding site elimination into their daily routines. This active participation and self-management culture contributes to the establishment of a long-term maintenance mechanism. Matatang village’s mosquito control practices, centered on environmental management and health education, exhibit notable sustainability and replicability.

Surveillance data revealed that Matatang village previously experienced high mosquito abundance, adversely affecting both residents’ daily lives and homestay tourism economies, garnering widespread support for mosquito control initiatives. CDC experts provided technical support (32) by assisting village leaders in formulating control strategies, conducting technical training for villagers and volunteers, and evaluating control effectiveness. Mosquito abundance assessment comprised adult and larval surveillance using methodologies comparable to other studies (33, 34). CO₂-baited mosquito light traps were employed to compensate for the poor attraction efficacy of conventional light traps for *Ae. albopictus* (35). The larval surveillance method was adapted from the Breteau Index (36), with modification to include all mosquito larvae rather than solely *Aedes* species. In Matatang village, the monitored *Cx. quinquefasciatus* and *Cx. tritaeniorhynchus* are the primary vectors of Japanese encephalitis (JE), while *Ae. albopictus* and *An. sinensis* are the vectors for dengue fever and malaria, respectively. However, mosquitoes native to Zhejiang Province do not naturally carry the dengue virus or malaria parasites. A significant reduction in mosquito density could help prevent JE as well as local transmission triggered by imported cases of dengue fever and malaria. Provincial, municipal, and county experts regularly conducted health education lectures covering mosquito control, environmental sanitation, and healthy lifestyles, supplemented by door-to-door campaigns and on-site guidance to disseminate scientific control knowledge. To expand outreach, the village installed educational signage in public areas including green spaces and building walls. Volunteer activities and peer communication among villagers further promoted health knowledge dissemination. These educational interventions cultivated villagers’ health literacy and fundamental capacity for scientific mosquito control (37), enhancing intrinsic motivation for health maintenance and fostering a supportive community atmosphere. Consequently, villagers actively participated in control activities, experiencing firsthand the benefits of scientific approaches, with early adopters becoming advocates and disseminators of control knowledge.

Matatang village’s control model exemplifies the “One Health” concept through its interdisciplinary collaboration involving agricultural and medical sciences, multi-sectoral coordination, and cross-regional cooperation (38, 39). The village’s mosquito control activities embody Integrated Vector Management (IVM) principles—a rational decision-making process for optimal resource allocation in vector control (40). IVM emphasizes both chemical and non-chemical methods, surveillance-based decision making, and combines advocacy, social mobilization, and legislative support with capacity building (infrastructure development, financial and human resources) (41). The village mobilized residents to conduct comprehensive environmental sanitation campaigns across residential and public areas, including: cleanup of over 150 waste-accumulated public areas, dredging of 1.5 km of waterways, removal of aquatic plants from over 20 households, disposal of approximately 50 tons of waste, and full connection of domestic sewage to municipal pipelines. These efforts resolved persistent environmental issues related to family workshops, livestock breeding, and waste recycling operations, significantly improving village conditions. Additional measures included installation of Solar-powered mosquito light traps, introduction of larvivorous fish in waterways, and application of slow-release larvicides in underground pipelines—collectively targeting breeding site reduction. The comprehensive control measures, particularly environmental remediation, yielded noticeable results in the second half of 2018, with mosquito abundance progressively declining to low levels that persisted through 2021. Although mean abundance indices differed between 2018 and 2021, the variation lacked statistical significance, potentially due to small sample sizes (42), high within-group data variability (large standard deviations) (43), and insufficient statistical power to detect true differences. However, significant differences in 2021 abundance indices between Matatang village and control villages confirmed the sustained effectiveness of the intervention. Environmental remediation and breeding site elimination remain the cornerstone and most challenging aspects of sustainable mosquito control (44). Thorough environmental management requires certain financial investment. For villages with limited or no funding sources, even maximizing environmental control efforts can significantly reduce mosquito abundance.

The survey results on villagers’ knowledge, behaviors and perceptions regarding mosquito control underscored the pivotal role of health education in improving health literacy. Through systematic health education and on-site guidance, Matatang village significantly enhanced residents’ awareness of mosquito breeding sites, demonstrating the efficacy of targeted interventions. However, both villages exhibited low knowledge rates regarding dengue fever—a discrepancy suggesting inadequate coverage of core information in current campaigns, potentially compromising prevention effectiveness. Alternatively, limited literacy levels may hinder comprehension of complex disease mechanisms (e.g., dengue transmission pathways). Nevertheless, villagers’ clear understanding of evidence-based mosquito control measures can offset their limited knowledge of vector-borne diseases. At the behavioral level, both villages relied on conventional chemical and physical methods (e.g., mosquito coils, bed nets). However, Matatang village showed significantly higher adoption rates of practices such as stagnant water elimination, ditch maintenance, and regular water changes for hydroponic plants. Notably, the control village predominantly depended on government-organized insecticide spraying, whereas Matatang residents exhibited greater acceptance of integrated approaches (e.g., environmental management, education campaigns). This shift implies that health education may have transformed villagers’ perception of governmental roles—from passive recipients to active participants. Differences in information acquisition further highlighted the need for diversified communication strategies. Matatang village reported higher reliance on lectures and interpersonal exchanges, suggesting that community-engaged education enhances knowledge penetration. We recommend integrating new media platforms (e.g., Douyin/TikTok) with offline activities to establish a multidimensional dissemination network for broader population coverage.

Satisfaction rates reflect villagers’ lived experiences with mosquito control outcomes, serving as a robust metric for perceived benefits and well-being (45). Mosquito management requires sustained efforts rather than one-time solutions. Residents of Matatang village reported significantly higher satisfaction rates with local mosquito control measures compared to the control village. The science-based interventions enhanced perceived benefits among villagers, where comprehensive environmental remediation not only effectively reduced mosquito abundance but also improved community esthetics. Over time, residents developed habitual practices (e.g., proactive breeding site removal), fostering a self-sustaining mechanism for long-term maintenance.

This study has several limitations. First, the small sample size (30 households per village, 60 questionnaires total), drawn from pre-existing village surveys, may limit statistical power and obscure occupational or age-related behavioral variations. Second, the cross-sectional design only captured 2021 maintenance-phase data, lacking longitudinal tracking of mosquito abundance changes from 2019 to 2020. This gap constrained our ability to assess the sustained effects of health education and environmental management. Additionally, the survey lacked pre-2018 historical data for comparison. Future studies should expand sample sizes and employ longitudinal designs to establish causal relationships and evaluate intervention sustainability.

## 5 Conclusion

The mosquito control efforts in Matatang village adhere to an environmentally friendly approach, prioritizing the elimination and modification of mosquito breeding sites through scientifically supported measures. By establishing a long-term mechanism centered on villager-led management and proactive maintenance, the initiative has not only improved the rural living environment but also achieved effective mosquito control. The optimal mosquito control outcomes were attributable to three synergistic determinants: evidence-based technical guidance from CDC personnel, robust coordination by village leadership, and active community engagement in intervention implementation. The sustained reduction in mosquito populations reflects an innovative exploration of rural mosquito control strategies, effectively addressing challenges such as limited health awareness among residents and the absence of sustainable management mechanisms. Supported by scientific expertise, multi-sectoral collaboration, and active community participation, Matatang village’s model plays a vital role in preventing mosquito-borne diseases and safeguarding public health. This experience offers valuable insights for replicable and scalable mosquito control practices in similar rural settings.

## Data Availability

The original contributions presented in the study are included in the article/supplementary material, further inquiries can be directed to the corresponding author.
